# Switching to Degludec From Other Basal Insulins Is Associated With Reduced Hypoglycemia Rates: A Prospective Study

**DOI:** 10.1210/jc.2019-01021

**Published:** 2019-08-09

**Authors:** Gian Paolo Fadini, Michael Feher, Troels Krarup Hansen, Harold W de Valk, Mette Marie Koefoed, Michael Wolden, Esther Zimmermann, Johan Jendle

**Affiliations:** 1 Department of Medicine, Division of Metabolic Diseases, University of Padova, Padova, Italy; 2 Beta Cell Diabetes Centre, Chelsea and Westminster Hospital, London, United Kingdom; 3 Department of Clinical and Experimental Medicine, University of Surrey, Guildford, United Kingdom; 4 Steno Diabetes Center Aarhus, Aarhus University Hospital, Aarhus, Denmark; 5 Department of Internal Medicine, University Medical Center Utrecht, CX Utrecht, Netherlands; 6 Novo Nordisk A/S, Søborg, Denmark; 7 Faculty of Medicine and Health, School of Medical Sciences, Örebro University, Örebro, Sweden

## Abstract

**Context:**

Observational studies of insulin degludec (degludec) with hypoglycemia events prospectively recorded are lacking.

**Objective:**

To evaluate the safety and effectiveness of degludec in patients with type 1 diabetes (T1D) or type 2 diabetes (T2D) switching from other basal insulins in routine care.

**Design:**

Results From Real-World Clinical Treatment With Tresiba^®^ was a multinational, multicenter, prospective, observational, single-arm study comprising a 4-week baseline period (preswitch basal insulin) and 12-month follow-up (degludec).

**Setting:**

Routine clinical practice.

**Patients or Other Participants:**

Insulin-treated patients (≥18 years) with T1D (n = 556) or T2D (n = 611) with treatment plans to initiate degludec.

**Interventions:**

Switching to degludec from other basal insulins.

**Main Outcome Measure:**

Change from baseline in number of overall hypoglycemic events recorded in patient diaries.

**Results:**

In T1D, the 12-month follow-up/baseline rate ratios (95% CI) of overall [0.80 (0.74 to 0.88)], nonsevere [0.83 (0.76 to 0.91)], severe [0.28 (0.14 to 0.56)], and nocturnal [0.61 (0.50 to 0.73)] hypoglycemia suggested significantly lower hypoglycemia rates with degludec (all *P*s < 0.001). At 12 months, HbA1c, fasting plasma glucose (FPG), and basal insulin dosage decreased significantly. Body weight increased, and treatment satisfaction improved significantly. In T2D, the hypoglycemia rate ratios were overall [0.46 (0.38 to 0.56)], nonsevere [0.53 (0.44 to 0.64)], and nocturnal [0.35 (0.20 to 0.62)] (all *P*s < 0.001; too few events for analysis of severe hypoglycemia). At 12 months, HbA1c and FPG decreased significantly. Body weight and insulin dosages remained unchanged, and treatment satisfaction was significantly improved.

**Conclusions:**

In a routine clinical care setting, switching to degludec from other basal insulins was associated with significantly lower rates of hypoglycemia, improved glycemic control, and treatment satisfaction in patients with T1D or T2D.

## Teaser

The Results From Real-World Clinical Treatment With Tresiba study demonstrated that use of degludec was associated with significantly lower hypoglycemia rates and better glycemic control in patients switching from other basal insulins.

Hypoglycemia is a common treatment-related event among patients with type 1 diabetes (T1D) or type 2 diabetes (T2D) treated with insulin and is often a key barrier to obtaining good glycemic control (1, 2). Additionally, hypoglycemia may adversely affect physical, mental, and social functioning, compromises work and leisure activities, and may lead to delays in treatment intensification (3–7). Therefore, reducing the risk of insulin-induced hypoglycemic events is essential in the management of diabetes.

Insulin degludec (degludec) is a basal insulin analog with an ultralong duration of action >42 hours at steady state and a lower day-to-day variability in blood glucose (BG)-lowering effect compared with insulin glargine 100 U/mL (glargine U100) (8, 9) and 300 U/mL (glargine U300) (10). In addition, treat-to-target, randomized controlled trials (RCTs) have demonstrated that degludec is associated with a reduced risk of hypoglycemia compared with other basal insulin analogs, at equivalent glycemic control, for patients with either T1D or T2D (11–15).

RCTs are considered the gold standard in providing evidence of drug efficacy and safety under controlled trial conditions in a specific patient population (16, 17). However, other sources of data are necessary to establish the effectiveness and long-term safety of drug treatments in real-world clinical practice and in a broader patient population (16, 17). Therefore, the Results From Real-World Clinical Treatment With Tresiba^®^ (ReFLeCT) study was designed to explore the safety and effectiveness of switching patients from other basal insulins to degludec over a 12-month period via a prospective study design. While ReFLeCT was ongoing, treatment with degludec was also being evaluated in other real-world studies with both retrospective and prospective study designs (18–29). These real-world studies demonstrated that degludec was associated with improved glycemic control and reduced hypoglycemia rates compared with previous basal insulin treatment. However, none of these studies prospectively collected hypoglycemia data from patient diaries and were thus limited by the bias of patients’ recollection of previous events in electronic medical records. ReFLeCT was established as an observational study that collected hypoglycemic event data prospectively by using patient diaries and evaluated patients’ perspectives on treatment with degludec.

The aims of the ReFLeCT study were to evaluate the clinical safety and effectiveness of switching to degludec from other basal insulins in routine clinical care of insulin-treated adults with T1D or T2D attending multiple diabetes centers across Europe.

## Materials and Methods

### Study design and population

ReFLeCT was a multicenter, prospective, observational study (NCT02392117) conducted at 153 sites across seven European countries (Denmark, the Netherlands, Spain, Sweden, Switzerland, Italy, and the United Kingdom) between March 2015 and March 2018. No stratification by country was used for recruitment.

Patients were eligible for inclusion in this study if they were aged ≥18 years, they had a clinical diagnosis of T1D or T2D, they were already on insulin, and their physician decided that they should switch to degludec treatment. The final decision to initiate the patient on degludec occurred within routine clinical practice. Patients were excluded from this study if they had previously been treated with degludec, had known or suspected hypersensitivity to degludec or any of the excipients as listed in the summary of product characteristics, or had mental incapacity, unwillingness, or language barriers precluding adequate understanding or cooperation (8).

**Figure 1.**
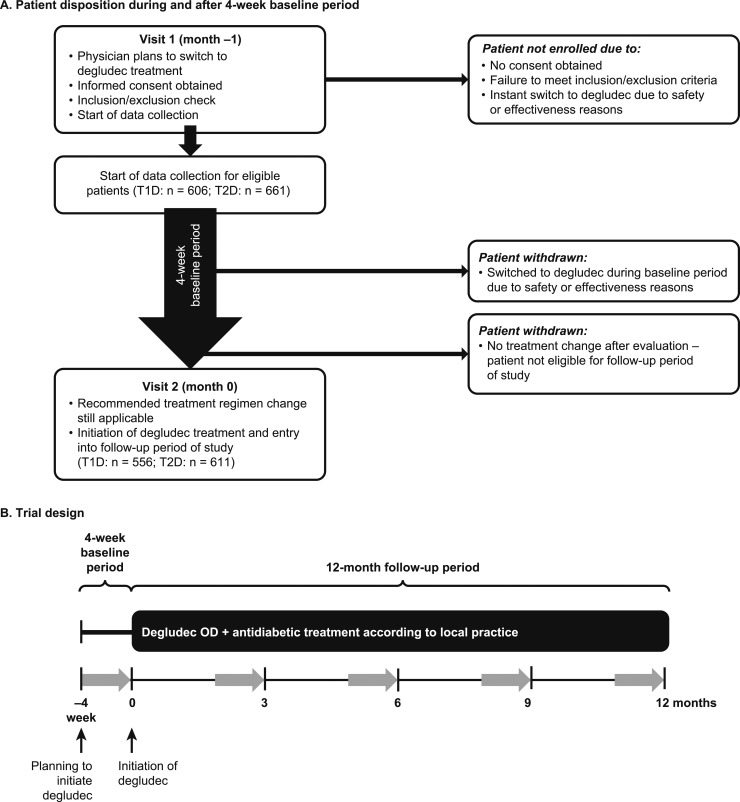
Schematic of trial design. (A) Patient disposition and (B) trial design. Gray arrows indicate 4-wk periods when hypoglycemic episodes will be recorded prospectively in patient diaries. Visits are defined as contact with the treating physician. OD, once daily.

This study consisted of a baseline period (∼4 weeks on patients’ current basal insulin regimen before the switch to degludec) and a follow-up period [≤12 months (±45 days) after switching to degludec] (Fig. 1). At the end of the 4-week baseline period, the treating physicians reassessed patients and confirmed the decision to switch to degludec. Patients who switched to degludec before the end of the 4-week baseline period (for safety or effectiveness reasons) or whose physician decided not to switch them to degludec were not eligible to continue in the study (Fig. 1). Patients were instructed on the local degludec label as per usual practice. Other than their basal insulin, patients could continue their other background glucose-lowering therapies throughout the study. Change in dosage, dose interval, and add-on or removal of bolus insulin and other antihyperglycemic drugs was at the discretion of the treating physician. If patients discontinued degludec during the 12-month follow-up period, they were withdrawn from the study at that time point.

Patients were considered hypoglycemia prone if they had any of the following: experienced at least one severe hypoglycemic event within the last year; moderate chronic renal failure, defined as an estimated glomerular filtration rate of 30 to 59 mL/min/1.73 m^2^ per the Chronic Kidney Disease Epidemiology Collaboration formula as specified by physician statement (or according to national reference definitions if they differ from the values stated); hypoglycemic symptom unawareness (history of impaired autonomic responses); for T1D, diabetes duration >15 years; for T2D, exposed to insulin for >5 years.

Clinical, laboratory, and patient-reported outcome (PRO) data were collected as part of routine clinical practice in the following timeframes during the 12-month follow-up period: 0 months (+14 days), 3 months (±45 days), 6 months (±45 days), 9 months (±45 days), and 12 months (±45 days). Patients attended only visits that were part of routine clinical practice, and thus not all patients were expected to attend all visits. HbA1c, fasting plasma glucose (FPG), and body weight were collected as part of routine clinical practice, and the results of the most recent tests were recorded at each visit. The bolus insulin dose on the day before the visit and data on the flexibility in timing of doses were collected by the treating physician at each visit. The Diabetes Treatment Satisfaction Questionnaire status version (DTSQ-s) and the Short Form-36 version 2 (SF-36^®^ v2) health status survey were completed by patients before or at each visit.

All patients were provided with up to five study diaries; the first covered the 4-week baseline period (before switching to degludec) and the remaining covered the 4 weeks before each subsequent physician visit (months 3, 6, 9, and 12 after switching to degludec). Therefore, if patients attended a visit every 3 months, they could contribute four 4-week diaries during the 12-month follow-up period. The diaries collected day-by-day information on basal insulin dose (previous basal insulin in the baseline period and degludec throughout the 12-month follow-up period) and time of administration. To assess hypoglycemia, patients answered the question, “Did you have one or more hypos today?” If the answer was “yes,” more detailed information about the hypoglycemic event, such as date and time of event, if it was self-treated, symptoms experienced, BG value (if recorded), and resource use was captured.

Informed consent was obtained from all patients, in accordance with the requirements of the Declaration of Helsinki, before any study-related activities (30). Independent ethics committees and institutional review boards across the participating centers reviewed and approved this study.

### Endpoints

The primary endpoint was the change from baseline in the number of overall hypoglycemic events during the 12-month follow-up period. The number of hypoglycemic events was converted to rates per patient-year of exposure for analysis purposes. Overall hypoglycemia was defined as any event recorded as hypoglycemia in the diary irrespective of symptoms, BG measurement, or time of day. Secondary endpoints related to hypoglycemia included change from the baseline period in the number of severe, nonsevere, nocturnal, severe or BG-confirmed, and severe or BG-confirmed symptomatic hypoglycemic events. Severe hypoglycemia was defined, according to the American Diabetes Association definition, as an event necessitating the assistance of another person to actively administer carbohydrate, glucagon, or other corrective actions (31). Nonsevere hypoglycemia was defined as either an event with or without symptoms accompanied by a BG measurement ≤3.9 mmol/L (70 mg/dL) or an event with symptoms not accompanied by a BG measurement but assumed to be caused by a BG ≤3.9 mmol/L (70 mg/dL). Nocturnal hypoglycemia was defined as an event (either severe or nonsevere) occurring between 00:01 and 05:59 (both inclusive), regardless of whether the patient was awake or woken up. Severe or BG-confirmed hypoglycemia was defined as an event that was severe (necessitating third-party assistance) or confirmed by a BG of <3.1 mmol/L (56 mg/dL). Severe or BG-confirmed symptomatic hypoglycemia was defined as an event that was severe (necessitating third-party assistance) or confirmed by a BG of <3.1 mmol/L (56 mg/dL) with symptoms.

Other secondary endpoints included HbA1c, FPG, daily insulin dosage (total, basal, and bolus), body weight, health-related quality of life questionnaire scores (PROs: DTSQ-s and SF-36^®^ v2), and flexibility in timing of doses after 12 months of treatment with degludec.

The treatment satisfaction assessment from DTSQ-s consists of eight questions and provides a measure of how satisfied patients are with their treatment and how they perceive hypoglycemia in the real-world clinical setting (32). The questions related to treatment satisfaction are scored from 0 = very dissatisfied to 6 = very satisfied and are added up to produce a total score (range 0 to 36). Higher total scores indicate higher treatment satisfaction. The separate question regarding perceived frequency of hypoglycemia is rated differently, and a low score represents good perceived BG control (0 = never; 6 = most of the time). SF-36^®^ v2 is a validated questionnaire, comprised of 36 questions that yield scores for eight scales and summarized into two component scores (the Physical Component Score and the Mental Component Score) (33). Higher scores represent better health status, with a score of 50 being the mean for the general population.

### Statistical analyses

The sample size was determined on the basis of the primary endpoint under the following assumptions: a two-sided paired *t* test with a 5.0% significance level, a mean change of the paired differences of one hypoglycemic event per patient-year of exposure with a standard deviation of 7, and a 15% withdrawal or discontinuation rate. In total, 608 patients were to be enrolled for each diabetes type for ≥90% power in the primary analyses of the primary endpoint. Recruitment continued until the calculated sample size was reached.

All endpoints were analyzed separately for patients with T1D and T2D, based on the full analysis set, which included all patients who received at least one dose of degludec. Baseline characteristics and demographics were summarized via descriptive statistics, with mean (SD) or number (percentage) as appropriate. All patients contributed with all available data to the hypoglycemia analyses. The hypoglycemia endpoints were analyzed via negative-binomial regression specifying a log-transformed follow-up time offset term. Baseline covariates included period (before or after the switch to degludec), age, sex, HbA1c, diabetes duration, body mass index (BMI), and country. For T2D, additional covariates included bolus insulin (yes or no) and sulfonylurea or glinides (yes or no) because these concomitant medications could affect the rates of hypoglycemia. The data for the hypoglycemia endpoints were based on the diary-recorded hypoglycemic events within the diary collection periods. Not all patients filled in the diary for 28 consecutive days as instructed. Therefore, only diaries of 26 to 30 days’ duration were included in the analyses. In addition, the date of events recorded in the diary had to match the date of the event recorded on the hypoglycemia pages. The analyses compared the 12-month follow-up period (composed of up to four 4-week diaries per patient) with the 4-week baseline period.

All patients contributed with all available data to the HbA1c, FPG, body weight, and insulin dose analyses. The changes in HbA1c, FPG, body weight, and insulin dose were estimated via analysis of covariance with a mixed model for repeated measures based on the antedependence covariance structure. Covariates included visit, baseline value, age, sex, BMI, country, and diabetes duration. For T2D, additional time-varying covariates included bolus insulin (yes or no; omitted from the bolus insulin dose analysis), sulfonylurea or glinides (yes or no), glucagon-like peptide-1 receptor agonists (yes or no), and other antihyperglycemic drugs (yes or no). Patients who completed the study contributed to the DTSQ-s, SF-36^®^ v2, and flexibility in timing of dosage analyses. DTSQ-s (32) and SF-36^®^ v2 (33) were scored as per the questionnaire instructions, and the mean change from baseline (95% CI) was summarized. For DTSQ-s, a difference of more than half a standard deviation of the baseline score is considered a meaningful change (34). The number of patients who at 12 months were reported to have used the flexibility option for timing of doses were summarized using descriptive statistics with number and percentage. All statistical tests were two-sided, with a significance level of *P* < 0.05. All statistical analyses were performed using SAS, version 9.3 or higher (SAS Institute, Cary, NC).

### Sensitivity analyses of the primary endpoint (overall hypoglycemia)

Four sensitivity analyses were conducted to determine whether deviations in diary entry and missing data affected the primary endpoint results. Sensitivity analysis 1 was based on the 26- to 30-day diary period, where the dates of hypoglycemic events recorded on the hypoglycemia page fell within the diary period. Sensitivity analyses 2 and 3 were based on all diaries regardless of duration, where the dates of hypoglycemic events recorded on the hypoglycemia page matched the events in the diary exactly or fell within the diary period, respectively. Sensitivity analysis 4 was similar to the primary analysis but was based on patients who completed 12-months of follow-up and had a minimum of 23 diary days for each of the five diary periods.

## Results

### Baseline characteristics and demographics

**Figure 2.**
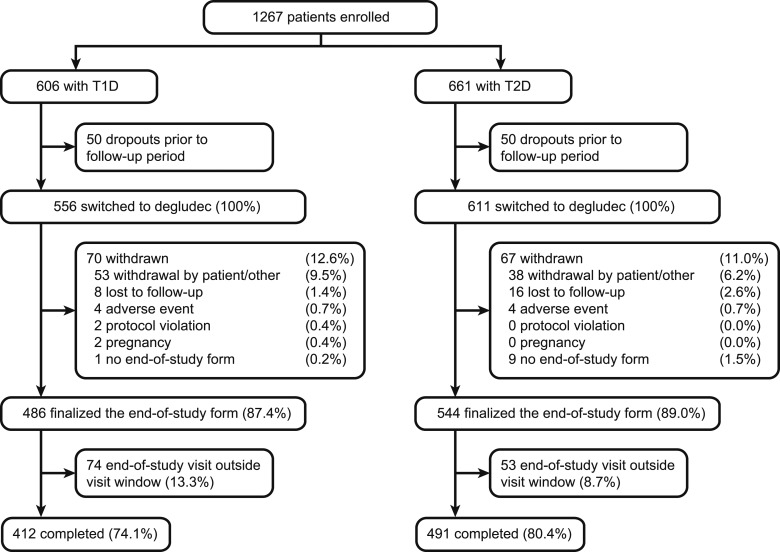
Patient disposition.

A total of 1267 patients were enrolled in the study (T1D, n = 606; T2D, n = 661). Out of these, 50 patients did not enter the follow-up period in both T1D and T2D, because either the treating physician did not confirm the switch to degludec or patients had switched to degludec during the baseline period, yielding 556 patients in the T1D group and 611 patients in the T2D group completing the baseline period and switching to degludec (Fig. 2). The baseline characteristics of the 50 patients in each group who did not enter the follow-up period were similar to those of the patients who entered the follow-up period.

After degludec initiation, 70 patients in the T1D group and 67 patients in the T2D group withdrew during follow-up. The majority of these withdrawals were due to “withdrawal by the patient” or “other reasons” (9.5% in T1D and 6.2% in T2D) (Fig. 2), with the most common explanations in both categories being diary burden, intensification with pump or combination products, and switch back to previous treatment.

Baseline characteristics and demographics are presented in Table 1. Patients in the T1D group had a mean age of 47.4 years, mean duration of diabetes was 21.4 years, 55.8% were male, and mean HbA1c was 8.1%. At baseline, 107 (19.2%) patients had an HbA1c <7.0%. Patients in the T2D group had a mean age of 65.2 years, mean duration of diabetes was 18.0 years, 59.6% were male, and mean HbA1c was 8.2%. At baseline, 100 (16.4%) patients had an HbA1c <7.0%. In addition, a large proportion of patients were considered hypoglycemia prone (T1D, 68.2%; T2D, 56.6%), defined as at least one of a list of risk factors (Table 1). For T1D, the majority of patients were from the United Kingdom (27%) and Italy (27%), whereas for T2D the majority of the patients were from Italy (51%).

**Table 1.** Baseline Characteristics and Demographics

Characteristic	T1D	T2D
Full analysis set, n	556	611
Age, y	47.4 (15.7)	65.2 (9.6)
Female/male, %	44.2/55.8	40.4/59.6
Country, n (%)		
Denmark	25 (4.5)	29 (4.7)
Netherlands	54 (9.7)	66 (10.8)
Spain	77 (13.8)	46 (7.5)
Sweden	68 (12.2)	42 (6.9)
Switzerland	36 (6.5)	81 (13.3)
Italy	148 (26.6)	311 (50.9)
United Kingdom	148 (26.6)	36 (5.9)
Duration of diabetes, y	21.4 (13.5)	18.0 (9.5)
BMI, kg/m^2^	26.1 (4.7)	31.1 (6.3)
Body weight, kg	76.4 (15.6)	87.6 (19.6)
HbA1c, %	8.1 (1.3)	8.2 (1.4)
HbA1c <7.0%, n (%)	107 (19.2)	100 (16.4)
HbA1c ≥7.0–<7.5%, n (%)	90 (16.2)	81 (13.3)
HbA1c ≥7.5–<8.5%, n (%)	174 (31.3)	217 (35.5)
HbA1c ≥8.5–<9.5%, n (%)	111 (20.0)	120 (19.6)
HbA1c ≥9.5%, n (%)	74 (13.3)	93 (15.2)
FPG, mmol/L	8.8 (3.9)	9.0 (3.1)
FPG, mg/dL^*a*^	158.4 (70.2)	162.0 (55.8)
Antidiabetic therapies at baseline		
Basal insulin before switch, n (%)		
Insulin glargine	355 (63.8)	361 (59.1)
Insulin detemir	126 (22.7)	127 (20.8)
Premix insulins	9 (1.6)	32 (5.2)
Other or missing	66 (11.9)	91 (14.9)
Metformin	41 (7.4)	307 (50.2)
SGLT-2 inhibitors	4 (0.7)	66 (10.8)
GLP-1 receptor agonists	4 (0.7)	64 (10.5)
DPP-4 inhibitors	2 (0.4)	49 (8.0)
Sulfonylureas	0 (0.0)	41 (6.7)
Thiazolidinediones	1 (0.2)	8 (1.3)
Total daily dose of basal insulin, U/d	25.0 (14.1)	37.5 (33.9)
Total daily dose of bolus insulin, U/d	27.3 (16.9)	38.9 (31.7)
Proportion on bolus insulin, n (%)	508 (91.4)	384 (62.8)
Time of basal insulin injections, n (%)		
Once-daily dosing regimen		
Morning	67 (12.1)	82 (13.4)
Evening	319 (57.4)	436 (71.4)
Twice-daily dosing regimen (morning and evening)	170 (30.6)	93 (15.2)
Hypoglycemia-prone patients,^*b*^ n (%)	379 (68.2)	346 (56.6)

Data are mean (SD) unless otherwise specified.

Abbreviations: DPP-4, dipeptidyl peptidase-4; GLP-1, glucagon-like peptide-1; SGLT-2, sodium-glucose cotransporter 2.

^a^ Calculated, not measured.

^b^ Defined as patients having any of the following: experienced at least one severe hypoglycemic event within the last year; moderate chronic renal failure, defined as estimated glomerular filtration rate 30–59 mL/min/1.73 m^2^ per Chronic Kidney Disease Epidemiology Collaboration formula, as specified by physician statement (or according to national reference definitions if they differ from the values stated); hypoglycemic symptom unawareness (history of impaired autonomic responses); for T1D, diabetes duration >15 y; for T2D, exposed to insulin for >5 y.

Basal insulins used by patients during the baseline period were diverse, reflecting standard clinical practice. The majority of patients switched from glargine (U100 or U300) to degludec in both the T1D group (63.8%) and the T2D group (59.1%) (Table 1). The most common reasons for switching to degludec were concern about hypoglycemia (64.6% in T1D, 36.2% in T2D) and need to improve BG control (63.9% in T1D, 73.3% in T2D). There were 8.7% of patients with T1D and 11.8% of patients with T2D who switched to degludec because their current regimen was too restrictive.

The proportion of patients using antidiabetic therapies during the 12-month follow-up period was similar to that of the baseline period (Table 2).

**Table 2.** Antidiabetic Therapies and HbA1c Levels During the 12-Mo Follow-Up Period

	T1D	T2D
Full analysis set, n	556	611
Metformin	41 (7.4)	314 (51.4)
SGLT-2 inhibitors	5 (0.9)	87 (14.2)
GLP-1 receptor agonists	4 (0.7)	74 (12.1)
DPP-4 inhibitors	2 (0.4)	54 (8.8)
Sulfonylureas	0 (0.0)	41 (6.7)
Thiazolidinediones	1 (0.2)	9 (1.5)
Total daily dose of basal insulin, U/d	22.8 (13.5)	35.9 (33.0)
Total daily dose of bolus insulin, U/d	23.8 (13.9)	38.3 (30.6)
Proportion on bolus insulin	501 (90.1)	378 (61.9)
HbA1c <7%	108 (19.4)	148 (24.2)
HbA1c <7.5%	193 (34.7)	270 (44.2)

Data are mean (%).

Abbreviations: DPP-4, dipeptidyl peptidase-4; GLP-1, glucagon-like peptide-1; SGLT-2, sodium-glucose cotransporter 2.

### Hypoglycemia

**Figure 3.**
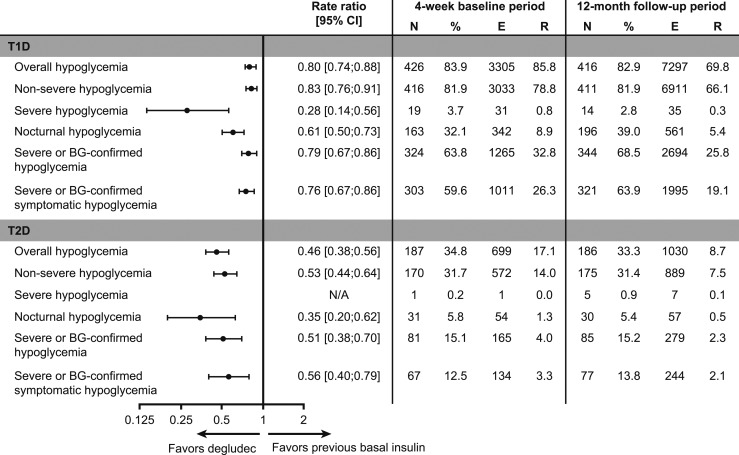
Hypoglycemia in patients with T1D or T2D during the 12-mo follow-up period vs the 4-wk baseline period. Hypoglycemia was analyzed via a negative binomial regression model controlled for period (pre/post), age, sex, HbA1c, diabetes duration, BMI, and country. For T2D, additional covariates included prandial insulin (yes or no) and sulfonylurea + glinides (yes or no). Total follow-up time (patient-years) for T1D was 38.5 and 104.5 for the 4-wk baseline period and the 12-mo follow-up period, respectively. Total follow-up time (patient-years) for T2D was 40.8 and 118.8 for the 4-wk baseline period and the 12-mo follow-up period, respectively. N/A, severe hypoglycemia could not be analyzed because of the low number of events. E, number of events; N, number of patients; R, number of events per patient-year of exposure.

**Figure 4.**
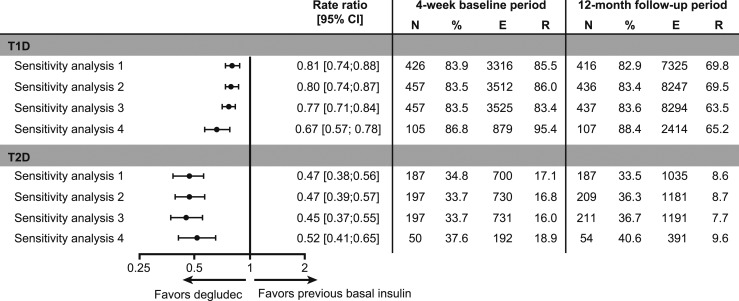
Sensitivity analyses in overall hypoglycemia in patients with T1D or T2D during the 12-mo follow-up period vs the 4-wk baseline period. Sensitivity analysis 1 is based on the 26- to 30-d diary period, where the dates of hypoglycemic events recorded on the hypoglycemia page fell within the diary period. Sensitivity analysis 2 is based on all diaries, where the dates of hypoglycemic events recorded on the hypoglycemia page matched the events in the diary exactly. Sensitivity analysis 3 is based on all diaries, where the dates of hypoglycemic events recorded on the hypoglycemia page fell within the diary period. Sensitivity analysis 4 is based on the 26- to 30-d diary period, where the dates of hypoglycemic events recorded on the hypoglycemia page matched the events in the diary exactly for patients with a minimum of 23 diary days for each diary period, who completed 12 mo observation. E, number of events; N, number of patients; R, estimated number of events per patient-year.

In patients with T1D, a significantly lower rate of overall hypoglycemia [rate ratio (RR) 0.80; 95% CI, 0.74 to 0.88] was observed during the 12-month follow-up period vs the 4-week baseline period (Fig. 3). Similarly, significantly lower rates of nonsevere (RR 0.83; 95% CI, 0.76 to 0.91), severe (RR 0.28; 95% CI, 0.14 to 0.56), nocturnal (RR 0.61; 95% CI, 0.50 to 0.73], severe or BG-confirmed (RR 0.79; 95% CI, 0.70 to 0.90), and severe or BG-confirmed symptomatic (RR 0.76; 95% CI, 0.67 to 0.86) hypoglycemia (all *P*s < 0.001) were also observed during the 12-month follow-up period vs the 4-week baseline period (Fig. 3). In patients with T2D, a significantly lower rate of overall hypoglycemia (RR 0.46; 95% CI, 0.38 to 0.56) was observed during the 12-month follow-up period vs the 4-week baseline period (Fig. 3). Similarly, significantly lower rates of nonsevere (RR 0.53; 95% CI, 0.44 to 0.64), nocturnal (RR 0.35; 95% CI, 0.20 to 0.62), severe or BG-confirmed (RR 0.51; 95% CI, 0.38 to 0.70), and severe or BG-confirmed symptomatic (RR 0.56; 95% CI, 0.40 to 0.79) hypoglycemia (all *P*s < 0.001) were observed during the 12-month follow-up period vs the 4-week baseline period (Fig. 3). Because of the low number of severe hypoglycemic events, no comparative statistics were performed (Fig. 3). All four sensitivity analyses were in line with the primary analysis, demonstrating the robustness of the primary analysis (Fig. 4).

### Glycemic control

**Figure 5.**
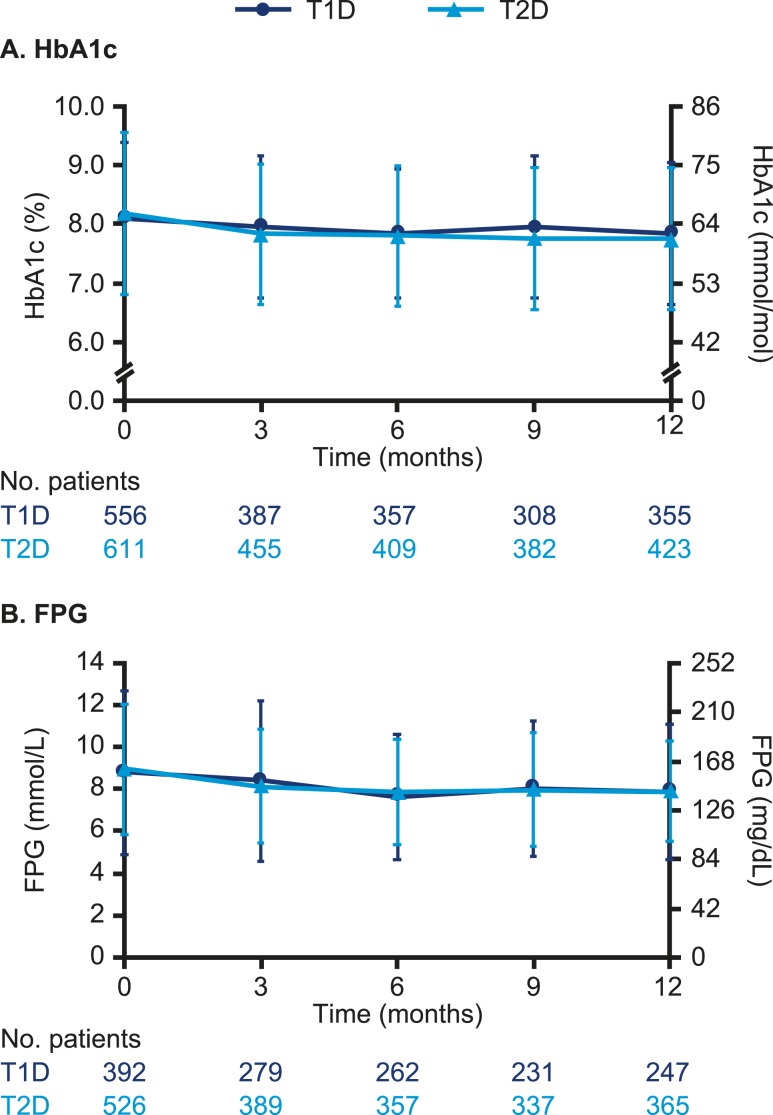
Observed mean (SD) (A) HbA1c and (B) FPG for patients with T1D or T2D after switch to degludec from other basal insulins.

In patients with T1D, the observed mean (SD) HbA1c decreased significantly from 8.1% (1.3) at baseline to 7.8% (1.2) at 12-month [estimated treatment difference (ETD) 12-month follow-up period vs 4-week baseline period, −0.15%; 95% CI, −0.23 to −0.07; *P* < 0.001] (Fig. 5A). The observed mean (SD) FPG decreased significantly from 8.8 mmol/L (3.9) [158.4 mg/dL (70.2)] to 7.9 mmol/L (3.2) [142.2 mg/dL (57.6)] (ETD −0.54 mmol/L; 95% CI, −0.95 to −0.14; −9.73 mg/dL; 95% CI, −17.12 to −2.52; *P* = 0.009) (Fig. 5B). In patients with T2D, the observed mean (SD) HbA1c decreased significantly from 8.2% (1.4) to 7.8% (1.2) (ETD −0.32%; 95% CI, −0.42 to −0.22; *P* < 0.001) (Fig. 5A). The observed mean (SD) FPG decreased significantly from 9.0 mmol/L (3.1) [162.0 mg/dL (55.8)] to 7.9 mmol/L (2.4) [142.2 mg/dL (43.2)] (ETD −0.84 mmol/L; 95% CI, −1.09 to −0.60; −15.14 mg/dL; 95% CI, −19.64 to −10.81; *P* < 0.001) (Fig. 5B).

### Insulin dose

Throughout the study, the majority of degludec injections were administered in the afternoon or evening (in particular 21:00 to 23:59) vs the morning by patients with T1D (afternoon or evening: month 3, 65.4%; month 6, 67.2%; month 9, 67.4%; month 12, 65.1%). A similar pattern was also observed for patients with T2D (afternoon or evening: month 3, 71.3%; month 6, 72.9%; month 9, 74.0%; month 12, 73.0%).

**Figure 6.**
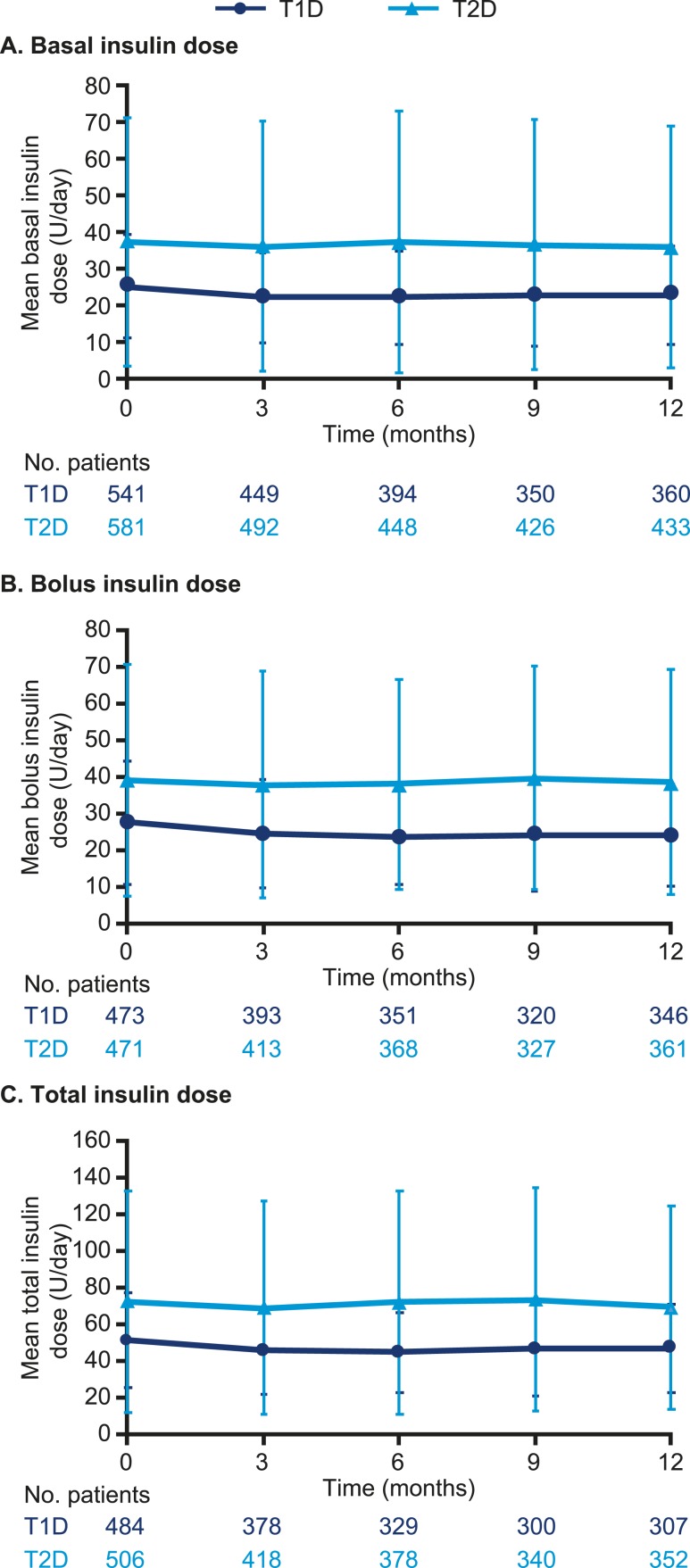
Observed mean (SD) (A) basal, (B) bolus, and (C) total insulin dose over time in patients with T1D or T2D after switch to degludec from other basal insulins.

In patients with T1D, the observed mean (SD) total daily basal insulin dose (U/day) decreased from 25.0 (14.1) (median 22.0; Q1 to Q3, 16.0 to 30.0) at baseline to 22.8 (13.5) (median 20.0; Q1 to Q3, 15.0 to 27.8) at 12 months (ETD −2.25 U/day; 95% CI, −2.85 to −1.66; *P* < 0.001) (Fig. 6A). The observed mean (SD) total daily bolus insulin dose (U/d) decreased from 27.3 (16.9) (median 24.0; Q1 to Q3, 17.0 to 33.0) at baseline to 23.8 (13.9) (median 20.5; Q1 to Q3, 15.0 to 30.0) at 12 months (ETD −3.19 U/d; 95% CI, −4.43 to −1.95; *P* < 0.001) (Fig. 6B). The observed mean (SD) total insulin dose (U/d) decreased from 51.1 (26.2) (median 47.0; Q1 to Q3, 35.0 to 62.0) at baseline to 46.8 (24.2) (median 41.0; Q1 to Q3, 31.0 to 55.5) at 12 months (ETD –5.39 U/d; 95% CI, –6.65 to –4.13; *P* < 0.001) (Fig. 6C). In patients with T2D, there was no significant difference in the observed mean (SD) total daily basal insulin dose at 12 months [35.9 U/d (33.0); median 26.1; Q1 to Q3, 18.0 to 42.0] vs baseline [37.5 (33.9); median 26.9; Q1 to Q3, 18.0 to 43.0; ETD −0.09 U/d; 95% CI, −1.48 to 1.30] (Fig. 6A). There was also no significant difference in the observed mean (SD) total daily bolus insulin dose at 12 months [38.3 U/d (30.6); median 30.0; Q1 to Q3, 19.0 to 46.0] vs baseline [38.9 (31.7); median 30.0; Q1 to Q3, 20.0 to 50.0; ETD 0.45 U/d; 95% CI, −1.60 to 2.50] (Fig. 6B). In addition, there was no significant difference in the observed mean (SD) total insulin dose at 12 months [69.3 U/d (55.6); median 53.4; Q1 to Q3, 34.0 to 86.2] vs baseline [72.4 (60.4); median 56.0; Q1 to Q3, 37.0 to 88.5; ETD 1.10 U/d; 95% CI, –1.65 to 3.84] (Fig. 6C). These insulin dose results at baseline and at 12 months did not change when adjusted for body weight (*i.e.,* U/kg/d; data not shown).

### Body weight

**Figure 7.**
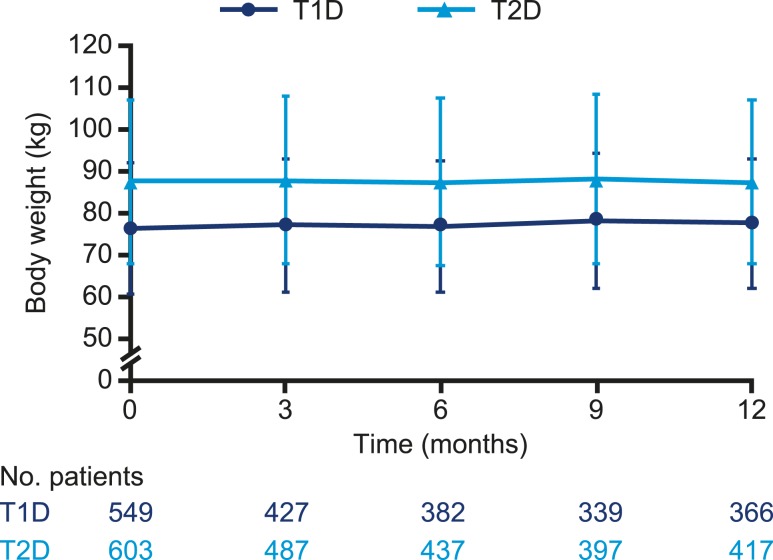
Observed mean (SD) body weight over time in patients with T1D or T2D after switch to degludec from other basal insulins.

The observed mean (SD) body weight significantly increased from 76.2 kg (15.6) at baseline to 77.5 kg (15.4) at 12 months (ETD 0.79 kg; 95% CI, 0.38 to 1.20; *P* < 0.001) (Fig. 7) in patients with T1D and was similar at baseline [87.4 kg (19.5)] and at 12 months [87.3 kg (19.6); ETD 0.09 kg; 95% CI, −0.39 to 0.57; *P* = 0.712] in patients with T2D.

### PROs

**Figure 8.**
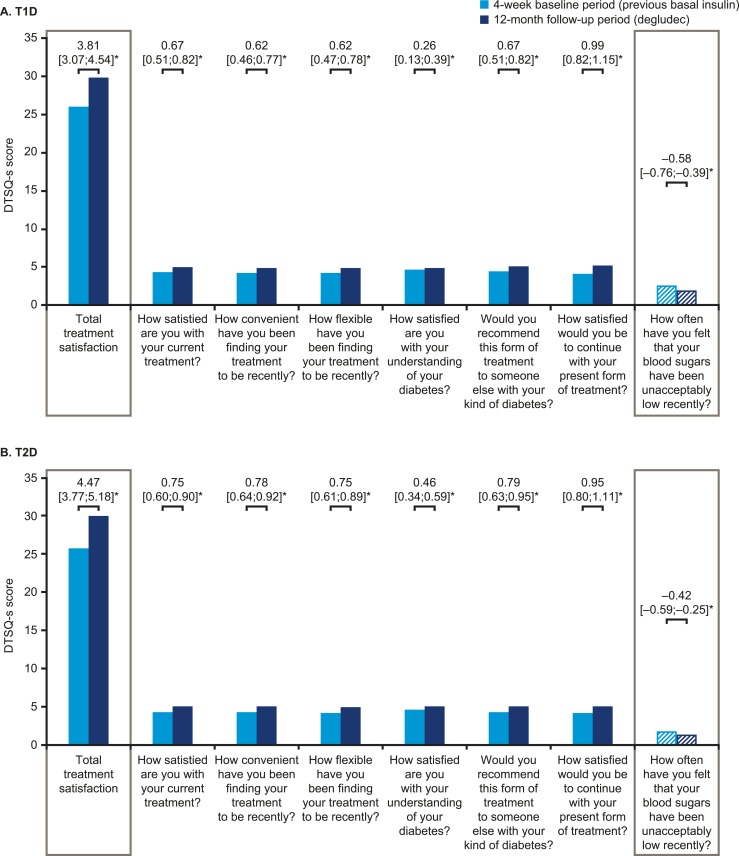
Mean treatment satisfaction in DTSQ-s in patients with (A) T1D or (B) T2D. Asterisks indicate significant differences. Data are changes between the 4-wk baseline period and the 12-mo follow-up period (95% CI). DTSQ-s scores range from 6 (very satisfied) to 0 (very dissatisfied). For the final question regarding perceived frequency of hypoglycemia, this question is treated separately from the other questions, and a low score represents good perceived blood glucose control.

**Figure 9.**
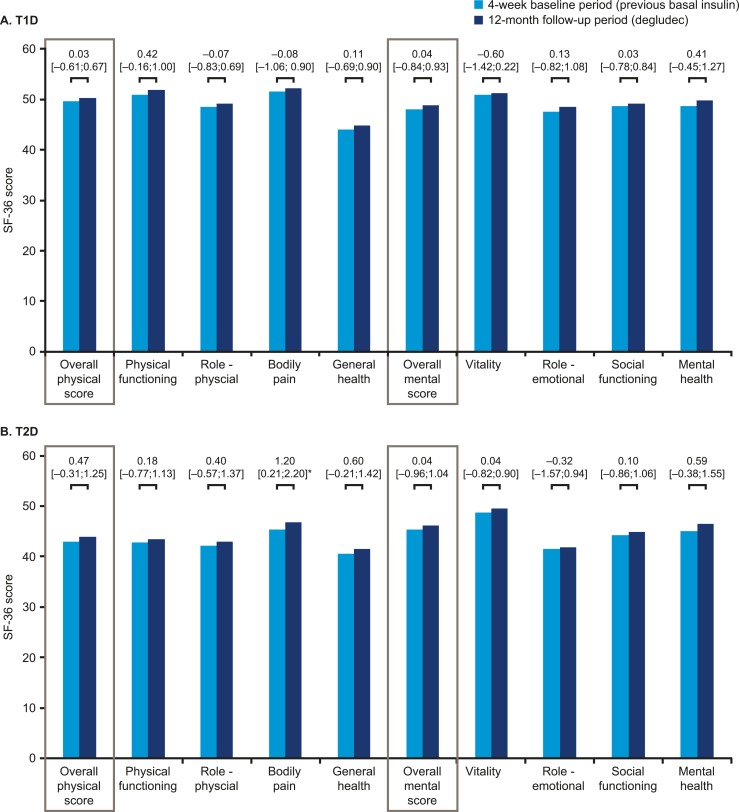
PROs in SF-36^®^ v2 for patients with (A) T1D or (B) T2D. Asterisks indicate significant differences. Data are changes between the 4-wk baseline period and the 12-mo follow-up period (95% CI).

The DTSQ-s overall total treatment satisfaction scores for both T1D and T2D significantly increased from baseline to 12 months, representing a clinically meaningful change (Fig. 8). Compared with their perspectives at baseline, patients were more satisfied with their treatment and perceived a lower frequency of hypoglycemic events after switching to degludec (Fig. 8). The results from the SF-36^®^ v2 survey demonstrated no significant differences between the baseline period and 12-month follow-up for either T1D or T2D, except for the bodily pain subdomain for T2D, which was significantly improved (Fig. 9). It should be noted that SF-36^®^ v2 is not a diabetes-specific quality-of-life questionnaire.

After 12 months 70/254 (27.6%) patients with T1D and 35/214 (16.4%) patients with T2D reported the need to take degludec at a different time at least once, and 53/254 (20.9%) patients with T1D and 30/214 (14.0%) patients with T2D used the specific flexibility option one or more times.

## Discussion

The ReFLeCT study demonstrated that switching to degludec from other basal insulins was associated with significant reductions in the rates of overall, nonsevere, nocturnal, severe or BG-confirmed, and severe or BG-confirmed symptomatic hypoglycemia, and improved glycemic control and treatment satisfaction in patients with T1D or T2D. Severe hypoglycemia was also significantly reduced from baseline in patients with T1D after they switched the basal insulin to degludec. This study was a long-term, prospective, observational study in a real-world setting evaluating the safety and effectiveness of degludec in routine clinical care.

ReFLeCT was a prospective study that collected hypoglycemia data from dedicated patient diaries. This design provided more accuracy in terms of data collection than studies based on patients’ recollections of events or studies based on events recorded in patients’ medical charts (22, 29). As expected, higher rates of hypoglycemia were observed in ReFLeCT compared with observational chart review or registry studies (22, 29). Yet, even with the use of diaries for data collection, diary fatigue leading to incomplete reporting and missing data are inherent problems that can bias the results. In ReFLeCT we observed that a small proportion of patients withdrew due to diary burden; however, at least two-thirds of patients at each visit had eligible diary data available. Furthermore, we mitigated this bias by ensuring that all available data were used in the analyses. Thus, ReFLeCT provides a more in-depth view of the burden of hypoglycemia experienced by patients that is typically not possible with retrospective chart reviews such as the EU-TREAT study, which also assessed degludec in routine clinical care (19).

The reduction in the rates of hypoglycemia observed after switching to degludec are in line with those of previous RCTs, where hypoglycemia rates were lower with degludec than with glargine U100 at similar glycemic levels, as well as other real-world studies (11, 13, 14, 19, 23). However, because of the nature of the study design and the lack of a comparator group (*i.e.,* a one-arm study), a treatment effect (regardless of the endpoint, level of glycemic control, reduction in hypoglycemia, or improvement in treatment satisfaction) cannot be isolated from a study effect. Furthermore, because antidiabetic treatments could be changed during the follow-up period, we cannot exclude the impact of these adjustments. In addition, it should be noted that the split between glargine U100 and U300 at baseline was not collected during the study, although, based on the study timeline, the majority of patients would have been using glargine U100. However, although the patients’ clinical situations in which the data were collected before and after the switch are somewhat different, the aim of the current study was to explore hypoglycemia rates, glucose control, and treatment satisfaction before and after switching to degludec in routine clinical care.

Because of its broad patient population, ReFLeCT may provide a more realistic estimate of the hypoglycemic burden of patients switching to degludec in a routine clinical setting compared with other studies. However, the hypoglycemia rates captured in ReFLeCT may, to some extent, be due to selection of the patients enrolled. It is plausible that patients with frequent hypoglycemia were overrepresented among those who agreed to participate in the study and whom their physician planned to initiate on degludec, which suggests that the findings are liable to regression to the mean. This was observed in patients with T1D, for 64.6% of whom hypoglycemia was the main reason for switching to degludec. This may also explain, at least in part, the modest reduction in HbA1c, because glycemic control may not be the primary focus for those patients and, in addition, approximately one-third of patients already had good glycemic control at study initiation.

Regardless of severity, hypoglycemic events can disrupt patients’ daily functioning and quality of life (35, 36), along with an added cost burden (37). In ReFLeCT, hypoglycemia and glycemic control were improved simultaneously. Combined with the improved treatment satisfaction, this study suggests that degludec has the potential to reduce these burdens where the need exists. Moreover, degludec offers the option for flexible timing of insulin doses, which, in addition to reducing patients’ fear of hypoglycemia, could also improve their adherence to treatment in the long term.

In summary, this study demonstrated that switching to degludec from other basal insulins was associated with significantly reduced rates of overall hypoglycemia with improved glycemic control and treatment satisfaction for patients with T1D or T2D in routine clinical practice.

## Abbreviations:

BG: blood glucose
BMI: body mass index
DTSQ-s: Diabetes Treatment Satisfaction Questionnaire status version
ETD: estimated treatment difference
FPG: fasting plasma glucose
PRO: patient-reported outcome
RCT: randomized controlled trial
ReFLeCT: Results From Real-World Clinical Treatment With Tresiba^®^
RR: rate ratio
SF-36^®^ v2: Short Form-36 version 2
T1D: type 1 diabetes
T2D: type 2 diabetes
